# Association of alcohol use and dietary lifestyle of commercial drivers during the COVID-19 pandemic in Nigeria

**DOI:** 10.1186/s42269-022-00885-y

**Published:** 2022-07-07

**Authors:** Great Iruoghene Edo, Laurine Chikodiri Nwosu

**Affiliations:** 1 Department of Petroleum Chemistry, Faculty of Science, Delta State University of Science & Technology, Ozoro, Nigeria; 2 Department of Chemistry, Faculty of Arts and Sciences, Cyprus International University, Nicosia, Cyprus; 3 Department of Health Care Organizations Management, Faculty of Health Sciences, Cyprus International University, Nicosia, Cyprus

**Keywords:** Nutritional status, Alcohol intake, Dietary pattern, Commercial motor drivers, Nigeria

## Abstract

**Background:**

Alcohol intake, particularly to cope up with stress and depression experienced by commercial drivers during the peak of the COVID-19 pandemic, is alarming as a rise in sales has been reported in certain countries during the quarantine. Alcoholism leads to malnutrition, either because those involved consume an insufficient amount of essential nutrients or because alcohol and its metabolism prevent the body from properly absorbing, digesting, and using those nutrients. This study was carried out to assess the association of alcohol use and dietary lifestyle of commercial motor drivers during the pandemic.

**Results:**

The anthropometric studies revealed that 69.5% of the respondents fall within the range of 18.5–24.49 indicating that they have normal weights. 63.5% reported daily consumption of alcohol, and 51% claimed that it does not affect their appetite, while 64.5% of the respondent stated that alcohol does not present them with any health problems. There was no significant association between the consumption of alcohol and nutritional status (*p* > 0.05), but a significant association between dietary lifestyle patterns and the nutritional status of participants was recorded (*p* < 0.05).

**Conclusion:**

It is, therefore, essential that commercial drivers are given adequate information and guidance on improving their dietary lifestyle.

## Background

A state of equilibrium in which nutrient intake and requirements are balanced is necessary for nutritional health. Two of the most important factors in achieving peak performance are nutrition and hydration. Alcohol is a poor nutrient source for a pre-game meal or hydration since it has been shown to slow one's ability to respond to an opponent or object 72 h after consumption (Kerksick et al. 2018). The assessment of nutritional status is a wide topic, and to be of clinical relevance, the ideal method should be able to detect whether the individual would have high morbidity and mortality in the absence of nutritional support (Bhattacharya et al. 2019). Unfortunately, disease and nutrition combine to lead to secondary malnutrition, or malnutrition may severely affect the underlying condition. Thus, patient outcomes are complex, and trying to construct nutritional effects on outcomes based on single parameters or simplistic models does not take into account the various interactive variables (Dipasquale et al. 2020).

As the intake of alcohol rises, the energy obtained from proteins, fats, and carbohydrates and the nutritional quality of the diet drops. Modifications in blood chemistry were linked to moderate and excessive alcohol intake. Recent statistics suggest that in emerging nations the use of recreational drugs has significantly risen, particularly alcohol, khats, and cigarettes (Wiers et al. 2021). Alcohol intake at high doses in particular tends to claim the lives of many people of Ede town in Southwestern Nigeria (Lasebikan et al. 2018). The global population of 12 years of age or older is projected to be 9 percent dependent on psychoactive drugs such as alcohol. A high level of alcohol consumption is associated with many mental disorders such as euphoria, hyperactivity, anorexia, insomnia, lewdness, and depression when they are shared with tobacco smoking (Ferreira et al. 2019). Alcohol also increases appetite and promotes extra calories that the body does not need (Stanton et al. 2020).

In a comprehensive review, evidence shows that moderate intake of alcohol was linked with reduced cardiovascular disease mortality, while heavy drinking leads to higher mortality compared to nondrinkers (Chiva-Blanch and Badimon 2019). Different studies and reviews have consistently found that consumption of 1–6 drinks a day was associated with a 20–50% lower risk of cardiovascular disease. Moderate alcohol intake has also been associated with a 20–30% reduced risk of stroke and other cardiovascular diseases and a 20% reduced risk of sudden death (Degerud et al. 2021). The pattern of drinking has distinct health implications regardless of the total consumption of alcohol. For example, beer bingeing was associated with increased all-cause mortality, death from external causes, and fatal myocardial infarction irrespective of total average beer, wine, and spirit intake (Ilic et al. 2018). Binge drinking may also partially explain the increase in CVD mortality, and the relationship between alcohol intake, CVD, and all-cause mortality is therefore likely to differ among a population where binge drinking and spirit consumption are common as compared to a population where moderate drinking of wine is predominant (Chiva-Blanch and Badimon 2019). In the past few decades, many studies particularly focused on diet modification as a key determinant in the development of cardiovascular diseases and found that an association between the intake of individual’s food or nutrient has a risk of cardiovascular disease. Consequently, the analysis of dietary patterns has been increasingly used in nutritional epidemiology taking into account the combined effect of food and potentially facilitating nutritional recommendation (Srour et al. 2019) (Yu et al. 2018). Among the sparsely reported data, commercial vehicle drivers have a high risk of chronic diseases, particularly diabetes mellitus, hypertension, and premature heart disease as compared to the general population and other occupational cohorts (Modjadji et al. 2022). There have been some reports evaluating relationships between obesity and factors within the Commercial Motor Vehicles (CMV) drivers population (Lavallière et al. 2020). Obese CMV drivers may also be prone to crashes, with a significantly higher crash rate (more than two times) compared with non-obese CMV drivers (Bschaden et al. 2019). This study was carried out to assess the association of alcohol use and dietary lifestyle of commercial motor drivers during the pandemic.

## Methods

### Study design

A descriptive and cross-sectional design was carried out.

#### Description of the study area

The study was conducted in Ogun state, Southwestern Nigeria. The state was established on February 3, 1976, from the Old Western State and covers 16 762 km^2^. The geographical location of the state makes it accessible to the economically developed regions of Nigeria. The state capital, Abeokuta, is about 103 and 70 km by road from Lagos and Ibadan, respectively. Other notable urban centers in the state, such as Ota, are about 17 km from Lagos, while Sagamu and Ijebu ode are about 65 and 75 km from Lagos, respectively, and 102 and 70 km from Ibadan by road. The geographical placement of Ogun State has made it a “gateway” to Nigeria from other coastal West African countries like Benin Republic, Sierra Leone, and Liberia. Ogun State's connection to the neighboring international community and much more developed Nigerian states strengthens its trade links and provides tremendous opportunities for growth and development. Because of its geographical location, the state has been able to attract and retain both foreign nationals and people from other Nigerian ethnic groups who find the state's various centers (especially urban areas) conducive to living and investment opportunities. The state comprises six ethnic groups, namely the Egba, the Ijebu, the Remo, the Egbado, the Awori, and Egun. Yoruba is the language of most people in Ogun, although this is nevertheless divided into several dialects.

The major garages that were worked on include Asero, Ita-oshin, Kuto, Lafenwa, Tipper, Ipara, Goro, Igbogbo.

### Study population

This comprises all commercial drivers in the selected motor—parks in Ogun state.

#### Sampling technique/procedures

A random sampling technique was used in this study to select registered drivers at the major garages in Ogun state. At the time of the study, there was a low population of commercial drivers due to low sales from transportation during the quarantine. Twenty-five drivers from eight major garages in the study area were selected. This was done to represent the total population of commercial motor drivers (CMD) in an unbiased manner.

#### Instruments used for data collection

The following instruments were used in collecting data for the study:

- Height meter
- Bathroom weighing scale

#### Method of data collection

- Questionnaire
  - The socioeconomic and demographic information of the respondents was obtained using a well-structured questionnaire.
  - 24-h dietary recall—this was used to assess the food intake and the daily alcohol intake of the respondents.
  - Food Frequency Questionnaire (FFQ)—this was used to assess the food habit and consumption patterns.
  - Consumption pattern of alcohol—to assess the frequency of alcohol consumption of the respondent.
- Anthropometric measurement—height meter and bathroom weighing scale were used to take the height and weight of the respondent, respectively.
- Assessment of alcohol use disorder (AUD)—This was done using the Fifth Edition of the Diagnostic and Statistical Manual of Mental Disorders (DSM-5) (American Psychiatric Association 2013). The evaluation of AUD was systematically carried out by an expert in addiction medicine. Each of the eleven DSM-5 criteria that occurred during the 12 months as well as significant distress was analyzed. This enabled the calculation of several AUD criteria ranging from 0 to 11. The severity of AUD was rated as low, intermediate, and high depending on the number of positive criteria (cutoffs at 2, 4, and 6, respectively).

### Data analysis

Microsoft Excel was used to describe and analyze the variables obtained from data collection. Data from other sections of the questionnaire such as socio-demographic characteristics, nutritional status, and alcohol consumption patterns were analyzed using Statistical Package for Social Science (SPSS, version 20.0). The data from the 24-h dietary recalls were analyzed using Total Dietary Assessment (TDA) software, and the level of significance was set at *p* < 0.05.

## Results

### Socioeconomic and demographic information of the respondents

**Fig. 1:**
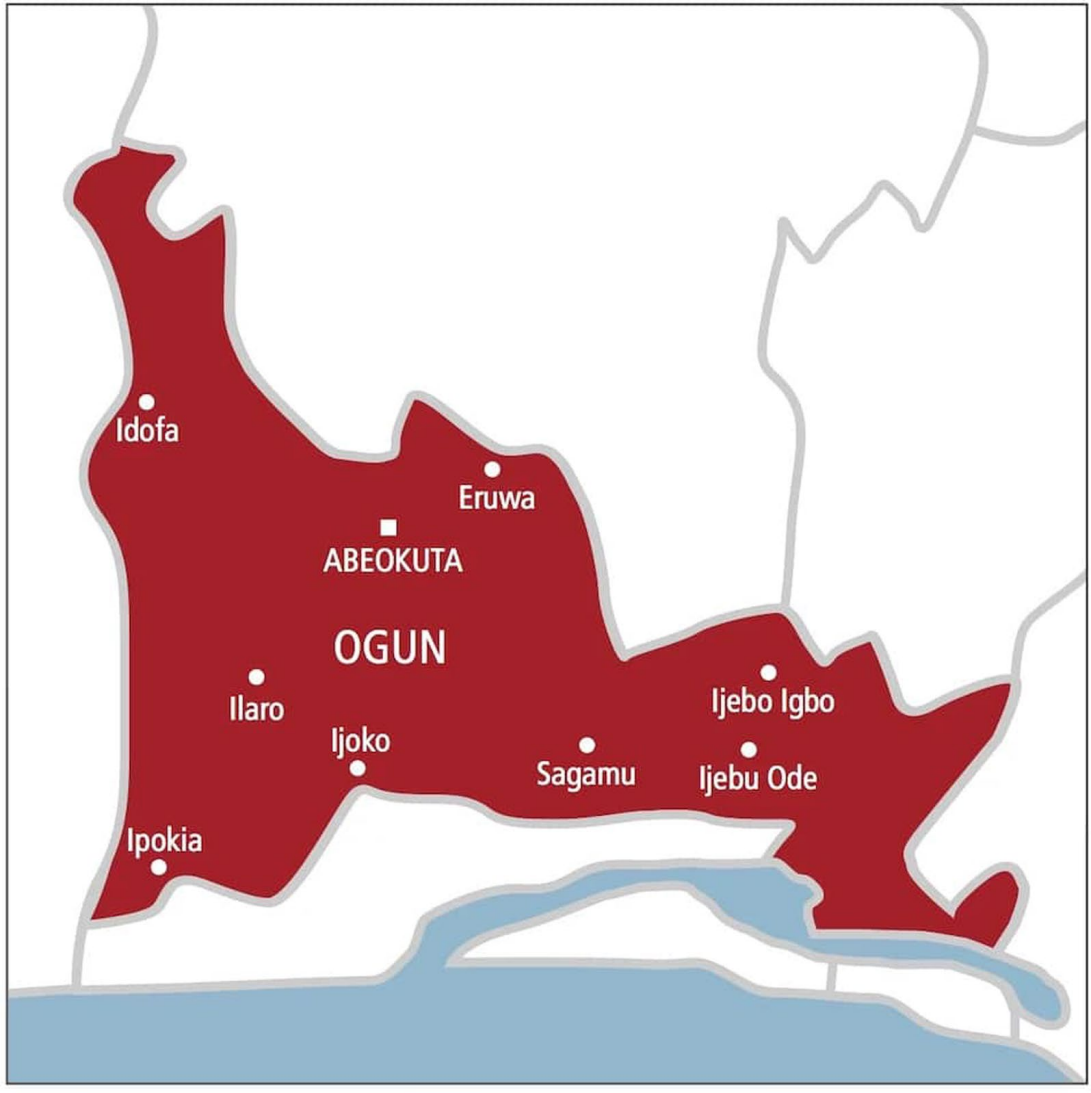
Geographical location of Ogun state, Nigeria

Table 1 shows the socioeconomic and demographic information of the respondents. From the study carried out, most of the participants were males, 35.5% of the respondents fell between the age group of 41–50 years, and 14.0% of respondents fell within the age range of 20–30 years. 28.0% of the respondents fall between the ranges of 31–40 of the age group, while 24.5% of the respondents are above 50 years of age. The highest family size fell within the range of 1–6 (77.5%), while 22.5% of the respondents fell within the range of 7–12. The majority (64.5%) of the respondent was from Ogun state, 16% from Oyo state, 9% from Osun state, 0.5% from Lagos state, 0.5% from Kwara state, 5% from Ekiti state, 3.5% from Ondo state, while the remaining 2% were from the eastern part of the country. From the study, it was reported that 93.0% of the respondents were married, 5% were single, and 2% were separated, while none were divorced or widowed. 36.5% of the respondents completed secondary school education, 28.5% completed primary school education, 8.5% completed tertiary education, and 5% of the respondents went to technical schools. From the estimated income result of the respondents, 44% of the respondents earned less than 5000 naira, 28% earned between 5001 and 15,000 naira, and 14.5% earned between 15,001 and 25,000 naira, while only 2% earned between 250,001 and 50000 naira (Fig. 1).

**Table 1:** Socioeconomic and demographic information of respondents

Variables	Frequency	Percentage
*Gender*		
Male	157	78.5
Female	3	1.5
*Ages (years)*		
20–30	28	14.0
31–40	56	28.0
41–50	67	33.5
> 50	49	24.5
*Family size*		
1–6	155	77.5
7–12	45	22.5
*State of origin*		
Eboyin	1	0.5
Edo	1	0.5
Ekiti	10	5.0
Kwara	1	0.5
Lagos	1	0.5
Ogun	129	64.5
Ondo	7	3.5
Osun	18	9.0
Oyo	32	16.0
*Marital status*		
Single	9	5.0
Married	187	93.0
Separated	4	2.0
Divorced	–	–
Widowed	–	–
*Level of Education*		
Primary completed	57	28.5
Primary not completed	28	14.0
Secondary completed	73	36.5
Secondary not completed	10	5.0
Tertiary completed	17	8.5
Tertiary not completed	10	5.0
Others	5	2.5
*Estimated income (Naira)*		
< 5000	88	44.0
#5001–15,000	56	28.0
#15,001–25,000	29	14.5
#250,001–50,000	4	2.0
#50,001–75,000	–	–
#75,001 and above	–	–

### Body mass index classification of the respondents

**Fig. 2:**
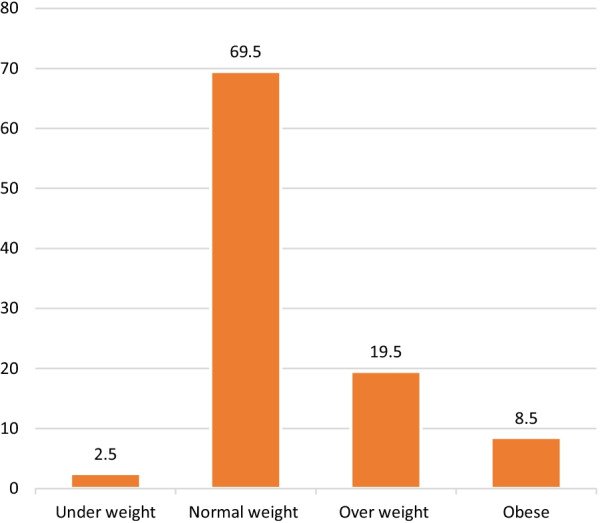
BMI classification of the respondents

Figure 2 shows the result of the anthropometric status of the respondents. WHO classification of body mass index was used in the classification of the anthropometric measurement of the respondents. 2.5% of the respondents were underweight falling between the range of less than 18.49 of the body mass index category, 69.5% of the respondents fell between the range of 18.5–24.49 indicating normal weight, 19.5% of the respondents fell between the range of 24.5–29.99 which indicate overweight, and 8.5% of the respondents fell under 30 and above which indicate obesity.

### Consumption pattern of respondents

Table 2 shows the consumption pattern of the respondents, 98% do not consume sorghum, and 43.5% consume semolina 1–3 days/week, while 81.5% consume rice 4–7 times/week. In the tuber and root group, 88% do not consume cocoyam, and 67% consume *Fufu* 1–3 times/week (Fufu contains 398 cal per 240 g serving. This serving contains 7.2 g of fat, 3.6 g of protein, and 81 g of carbohydrate. It is high in potassium and low in cholesterol), while 58.8% consume *Eba* 4–7 times/week. (It is a cooked starchy food made from dried grated cassava (manioc) flour, also known as garri.) In the legumes group, 96% do not consume soybeans, and 93% consume black-eyed peas 1–3 times/week, while 23.5% consume cowpea 4–7 days/week. In the meat group, 89.5% do not consume pork, and the highest consumed meat taken 1–3 times/week was kidney (41%), while fish (73.5%) was most consumed 4–7 times/week. In the vegetable group, 94.5% of respondents do not consume cabbage, and 42% take spinach 1–3 times/week, while 87% consume tomatoes 4–7 days/week. In the diary group, 84% do not consume yogurt, and 57% consume milk 1–3 times/week, while 15% of the respondents consume milk 4–7 times/week.

**Table 2:** Consumption pattern of respondents

Food groups	Never	1-3 days/week	4-7 days/week
*Cereals and grains*			
Maize pap	85(42.5%)	71(35.5%)	44(22%)
Cornflakes	193(69.5%)	1(0.5%)	4(2%)
Rice	8(4%)	29(14.5%)	163(81.5%)
Wheat bread	135(67.5%)	57(28.5%)	8(4%)
Semolina	101(50.5%)	87(43.5%)	12(6%)
Pasta	139(69.5%)	56(28%)	5(2.5%)
Sorghum	196(98%)	4(2%)	–
*Tubers and root vegetables*			
*Eba*	26(13%)	57(28.5%)	117(58.8%)
*Fufu*	44(22.0%)	134(67%)	22(11%)
Yam	70(35%)	124(62%)	6(3%)
Cocoyam	176(88%)	24(12%)	–
Potato	159(79.5%)	41(20.5%)	–
*Legumes*			
Groundnuts	103(51.5%)	71(35.5%)	26(17.5%)
Cowpea	97(48.5%)	56(28%)	47(23.5%)
Green peas	84(42%)	85(42.5%)	31(15.5%)
Black-eyed peas	85(42.5%)	107(93%)	14(7%)
Soybeans	192(96%)	8(4%)	–
*Meat groups*			
Lean meat	34(17%)	57(28.5%)	109(54.5%)
Liver	105(52.5%)	78(38%)	17(8.5%)
Kidney	100(50%)	82(41%)	18(9%)
Chicken	124(62%)	73(36.5%)	3(1.5%)
Turkey	130(65%)	69(34.5%)	16(8%)
Pork	179(89.5%)	17(8.5%)	4(2%)
Fish	9(4.5%)	50(25%)	141(73.5%)
*Vegetables*			
Tomatoes	16(8.0%)	10(5%)	174(87%)
Okra	124(62%)	66(33%)	10(5%)
Carrot	159(79.5%)	41(20.5%)	–
Cabbage	189(94.5%)	6(3%)	5(2.5%)
Cucumber	123(61.5%)	69(34.5%)	8(4%)
Spinach	93(46%)	86(42%)	21(11.5%)
*Diary group*			
Milk	56(28%)	114(57%)	30(15%)
Cheese	170(85%)	23(11.5%)	7(3.5%)
Yoghurt	168(84%)	24(12%)	8(4%)

### Quantity of alcohol consumed by respondents

Table 3 represents the quantity of alcohol consumed by respondents. 15% of the respondents consume 33, 36 bottles are consumed in a week, and 1.5% consume 33 cl, while 11.55% consume 60 cl. 14% of the respondents consume 33 cl of small stout, and 36 bottles are consumed in total by the respondents weekly. 11% of the respondents consume 60 cl of big stout, and a total of 11 bottles are consumed weekly. 19.5% of the respondents consume 36 bottles of origin, 8% consume 33 cl, and 11.5% consume 60 cl of origin. 8.5% of the respondents consume 60 cl of harp, and 27 bottles are consumed weekly. 40% consume trophy, and 11.5% consume 50 cl, while 28.5% consume 60 cl, and a total of 166 bottles are consumed a week. 11% of the respondents consume 60 cl of goldberg, and 75 bottles of trophy are consumed by the respondent per week. 12% consume 60 cl of gulder, with the consumption of 22 bottles in a week. 30.5% consume 268 sachets of Chelsea in 30 ml per week, and 22.5% of the respondents consume 30 ml of dry gin and a total sachet of 112 per week. 13% consume 14 33 cl can of bullet in a week. 17% reported the intake of palm wine, 4.5% consume 50 cl of palm wine, 4% consume 60 cl palm wine, and 7% consume 75 cl of palm wine, while 1.5% consume 1L of palm wine. 22 bottles of palm wine are consumed in a week.

**Table 3:** Quantity of alcohol consumed by respondents

Variables	Frequency *N* (%)	No of bottle consumed per week	Category of quantity consumed	Quantity consumed *N* (%)
33	30(15)	36	33 cl	3(1.5)
			60 cl	23(11.5)
Small stout	28(14)	36	33 cl	28(14)
Big stout	11(5.5)	11	60 cl	11(5.5)
Origin	39(19.5)	36	33 cl	16(8)
			60 cl	23(9.5)
Harp	17(8.5)	27	60 cl	17(8.5)
Trophy	80(40)	166	50 cl	23(11.5)
			60 cl	57(28.5)
Gulder	24(12)	27	60 cl	24(12)
Goldberg	22(11)	75	60 cl	22(11)
Star	12(6)	9	33 cl	2(1)
			60 cl	10(5)
Legend	15(7.5)	16	60 cl	15(7.5)
MacDowell	6(12)	4	60 cl	6(3)
Magic moment	3(1.5)	1	60 cl	3(1.5)
Vodka	3(1.5)	17	40 ml	3(1.5)
Chelsea	61(30.5)	268	30 ml	61(30.5)
Dry gin	43(21.5)	112	30 ml	43(18.5)
Smirnoff	2(1)	4	33 cl	2(1)
501	2(1)	1	75 cl	2(1)
Bullet	26(13)	14	33 cl	26(13)
Red bull	1(0.5)	2	33 cl	1(0.5)
Palm wine	34(17)	22	50 cl	9(4.5)
			60 cl	8(4)
			75 cl	14(7)
			1ltr	3(1.5)

### Mean energy and nutrient intake of the respondent

Table 4 shows the mean energy and nutrient intake of the respondents. The mean energy intake recorded was 2788.70 kcal which is more than the Recommended Dietary Allowance (RDA) of 2500 kcal, and the mean protein intake (115.51 g) was higher than the RDA of 56 g. The mean fat intake recorded (60.94) was lesser than the RDA of 102 g. The mean intake of carbohydrate reported was 402.63, which is more than the RDA of 325 g. 31.93 g was the mean dietary fiber intake of the respondents which is lesser than the RDA of 38.0 g. 3.6 mg was the mean intake of vitamin B1 which is higher than the recommended daily intake of 1.0 mg, mean intake for vitamin B2 was recorded as 1.09 mg which is lesser than the RDA of 1.2 mg, and 33.67 mg was the mean intake of vitamin B3 which is lesser than the RDA of 60 mg. Folate intake of the respondent was 464.12 µg which is higher than the RDA of 400.0 µg. 24.15 mg is the average vitamin C intake of the respondents which is lesser than the RDA of 100.0 mg. The mean intake of sodium is 1776.2 mg, which is lesser than the recommended daily intake of 2000.0 mg, and 1805.77 mg was the mean potassium intake of the respondents which is lesser than the RDA of 3500.0 mg. The mean calcium intake was 568.63 mg, which is lesser than the RDA of 1000.0 mg. 359.88 mg was the mean magnesium intake of the respondents which is more than the RDA of 300.0 mg. 50.12 mg was the mean iron intake of the respondents which is more than the RDA of 15.0 mg. Finally, 25.03 mg was the mean zinc intake of the respondents which is lesser than the RDA of 15.0 mg with a percentage RDA of 167%.

**Table 4:** Mean energy and nutrient intake of the respondents

Nutrient Analyzed	Mean intake	RDA	%RDA
Energy (kcal)	2788.70	2500	111.54
Protein(g)	115.51	56	206
Fat (g)	60.94	102	59.4
Carbohydrate (g)	407.63	325	124
Fiber (g)	31.93	38.0	84
Vitamin B1(mg)	3.56	1.0	296
Vitamin B2 (mg)	1.09	1.2	91
Vitamin B3(mg)	33.67	60	56
Folate (µg)	464.12	400	116
Vitamin C(mg)	24.15	100	24
Sodium(mg)	1776.27	2000	89
Potassium(mg)	1805.77	3500	52
Calcium(mg)	568.63	1000	57
Magnesium(mg)	359.88	300	102
Iron(mg)	50.12	15	201
Zinc(mg)	25.03	15	167

### Contribution of alcohol to the energy intake and nutrient intake of the respondents.

Table 5 shows the percentage contribution of alcohol to the energy intake and the nutrient intake of the respondents, and from the average calorie intake of 2788.70, alcohol contributes 450 kcal which is 16% of the average calorie intake of the respondents. Alcohol contributes 22.07% of the average carbohydrate intake of 407.63 contributing 25.5 g of carbohydrate. Beer contains a little amount of protein contributing 4.90% of the average protein intake of 115.51 g as 3.0 g, and 9.8% dietary fiber is contributed to the average dietary fiber of 31.93 contributing 3.0 to the dietary fiber of the respondent. Some micronutrients are also contributed to the nutrient intake of the respondent from alcohol, and alcohol contributes 60 mg of sodium and calcium to the average intake of 1776.27 and 568.63 mg, respectively, having a contribution of 3.37 and 24.50%, respectively. Alcohol contributes 24.50% of potassium contributed to the average intake of 1805.77 potassium intake of the respondents.

**Table 5:** Contribution of alcohol to the energy and nutrient intake of the respondent

Nutrient analyzed	Mean intake	Nutrient content of alcohol	% Contribution of alcohol to the nutrient intake
Energy (kcal)	2788.70	450	16.0
Protein(g)	115.51	3.0	4.90
Fat (g)	60.94	–	–
Carbohydrate (g)	407.63	25.5	22.07
Fiber (g)	31.93	3.0	9.30
Vitamin B1(mg)	3.56	0.3	8.42
Vitamin B2 (mg)	1.09	–	–
Vitamin B3(mg)	33.67	–	–
Folate (µg)	464.12	60	12.92
Vitamin C(mg)	24.15	–	
Sodium(mg)	1776.27	60	3.37
Potassium(mg)	1805.77	442.5	24.50
Calcium(mg)	568.63	60	10.55
Magnesium(mg)	359.88	–	–
Iron(mg)	50.12	–	–
Zinc(mg)	25.03	–	–

### Alcohol consumption habits of the respondents

Table 6 shows the alcohol consumption habit of the respondents. From the study carried out, 69% have taken alcohol, while 13% have not. Most of the respondents had an intermediate AUD (44%), while the least had low AUD (20%). 8% reported that alcohol is taken just on feast days, while 63.5% consume alcohol on a daily bases. 51% consuming alcohol claim that it does not affect their appetite, while 21% said that it does affect their appetite. 64.5% of the respondent also stated that alcohol does not present them with any health problems, while 8% stated that they are present with a health problem after the intake of alcohol, and among the 8%, 7% are presented with a headache, while 1% of the respondents is presented with fever. Alcohol replaces 11.5% of the respondents’ main meal, 7% of the respondent reported that it replaces dinner, and it replaces 4% of the respondent’s lunch, and 1.5% of the respondent’s breakfast. It was also recorded that 41% of the respondents have had alcohol in the past year, 67.5% have had alcohol in the last 30 days and 22.5% have had alcohol in the last 7 days. 36.5% consume alcohol 2–5 times per day, while 34.5% consume alcohol more than 5 times per day (Table 7).

**Table 6:** Alcohol consumption and lifestyle of the respondents

Variables	Options	Frequency	Percentage
Ever taken alcohol	Yes	138	69.0
	No	13	6.5
Alcohol use disorder (clinical interview)	Low	40	20.0
	Intermediate	88	44.0
	High	35	17.5
Is alcohol only taken on feast day	Yes	16	8.0
	No	127	63.5
Does alcohol affect your appetite	Yes	42	21.0
	No	101	51.0
If yes how?	Stimulate appetite	25	12.5
	Loss of appetite	17	8.5
Do you experience health related problem after intake of alcohol	Yes	13	6.5
	No	130	64.5
If yes how?	Fever	2	1.0
	Headache	14	7.0
Does alcohol replace your main meal	Yes	23	11.5
	No	120	60
If yes which meal	Breakfast	3	1.5
	Lunch	8	4.0
	Dinner	14	7.0
Alcohol intake in the past 1 year	Yes	82	41.0
	No	50	25.0
Alcohol intake in the last 30 days	Yes	135	67.5
	No	15	7.5
	Yes (1–7 days)	45	22.5
Alcohol intake in the last 7 days	Yes (1–5 days)	57	28.5
	Yes (2–3 days)	38	19.0
	No	20	10.0
	> 5 times	69	34.5
Alcohol intake per day	2–5 times	73	36.5
	0–7 days	25	12.5
Number of days involved in any form of physical activity in the last 30 days	8–15 days	30	15.0
	16–22 days	38	19.0
	23–29 days	72	36.0
	All 30 days	–

**Table 7:** Relationship between nutritional status and quantity of alcohol consumed by the respondents

Quantity consumed	Underweight	Normal	Overweight	Obese	Total	*P* value
33 33–60 cl	1(0.5%)	18(9.0%)	7(3.5%)	0(0%)	26(13%)	0.344
Small stout 33–60 cl	0(0%)	24(12%)	4(2%)	0(0%)	28(14%)	0.920
Big stout 33–60 cl	0(0%)	9(4.5%)	1(0.5%)	0(0%)	10(5%)	0.244
Origin 33–60 cl	2(1.0%)	26(13%)	8(4%)	2(1%)	38(18%)	0.138
Harp 33–60 cl	0(0%)	14(7%)	3(1.5%)	0(0%)	17(8.5%)	0.29
Trophy 33–60 cl	4(2%)	61(3.5%)	12(6%)	4(2%)	81(40.5%)	0.130
Gulder 33–60 cl	0(0%)	20(10%)	4(2%)	0(0%)	24(12%)	0.176
Goldberg 33–60 cl	0(0%)	12(6%)	6(3%)	3(1.5%)	21(10.5%)	0.57
Star 33–60 cl	0(0%)	10(5%)	2(1%)	0(0%)	12(6%)	0.448
Legend 33–60 cl	0(%)	8(4%)	3(1.5%)	1(1.5%)	12(6%)	0.68
Smirnoff 33–60 cl	0(0%)	2(1%)	0(0%)	0(0%)	2(1%)	0.074
Red bull 33–60 cl	0(0%)	1(0.5%)	0(%)	0(0%)	1(0.5%)	0.856
Palm wine 33–60 cl 75 cl–1ltr	1(0.5%) 1(0.5%)	10(5%) 7(3.5%)	4(2%) 6(3%)	0(%) 0(%)	15(7.5%) 14(7%)	0.712
Dry gin 30–40 ml	0(0%)	27(3.5%)	10(5%)	3(1.5%)	40(20%)	0.530
Vodka 33–60 cl	1(0.5%)	2(1%)	0(%)	0(0%)	3(1.5%)	0.74
MacDowell 33–60 cl	2(1%)	1(0.5%)	0(%)	0(0%)	3(1.5%)	0.009

### The relationship between nutritional status and the quantity of alcohol consumed by the respondents

From the study, 0.5% among the respondents that consume 33 are underweight, respondents with normal weight are 9 and 3.5% are overweight having a *p* value of 0.344. Therefore, this is not significant. Among the respondents that consume small stout, 12% have normal weight, and 2% are overweight having a *p* value of 0.92 which is not significant. Among the respondents that consume 33–60 cl of big stout, 4.5% have normal weight, and 0.5% are overweight with a *p* value of 0.244 showing insignificance between quantity consumed and nutritional status. 1% that consume 33–60 cl of origin are underweight, 13% have normal weight, 4% are overweight and 1% is obese having a *p* value of 0.138, and this indicates that it is insignificant. Out of the respondents that consume 33–60 cl of trophy, 2% are underweight, 3.5% have normal weight, 6% are overweight and 2% are obese having a *p* value of 0.13 which is not significant. 13.5% of the respondents that consume 30–40 ml of dry gin have a normal weight, 5% are overweight and 1.5% are obese having a *p* value of 0.530.

## Discussion

General observation suggests that many individuals with AUD do not consume a balanced diet. Moreover, where alcohol consumption is excessive, it may interfere with their ability to absorb and use the nutrients they do consume. From the age classification of the respondents, the majority fell between the ages of 31–40 years and 41–50 years. This is similar to a study conducted by (Ipingbemi 2008) on 600 commercial motor drivers in selected southwestern cities revealed that 35.5% were between 31 and 45 years and 27.3% between 45 and 60 years old with only 15% of the respondents over 60 years. However, it was noted that while some of the study areas had high population of elderly drivers like in Akure and Ado-Ekiti motor parks, others (particularly in urban cities) had a high number of youthful drivers which may be connected to the increased unemployment rate in the country causing young adults to move to urban cities in search of jobs. Over half of the sample respondents were men, confirming that commercial driving is primarily a male occupation. Despite the role of culture and the general perception, women have about commercial driving as a job reserved for thugs, and economic hardship is gradually introducing them into this job to assist in fending for their families.

More than 90% of the commercial drivers were married. The high percentage of married drivers was expected because of the level of education as majority of them had either primary or secondary school education as the highest form of learning and got married immediately after graduation. This finding is also similar to that of (Adepoju and Akinbode 2019). Many of commercial drivers are paid daily wages below 5000 naira which is similar to that of (Ipingbemi 2008) depending on the city and type of vehicle, which they consider meager. Such drivers stated that after allowing for vehicle maintenance, what is left is so meager that it cannot support them and their families.

This study shows that 8.5% of the respondents were obese, while 19.5% of the respondents were overweight indicating a low prevalence of obesity among the drivers. A similar study conducted by Gu et al. (2014) revealed a low prevalence of obesity among motor park workers (male) compared to overweight workers (15.4 and 29.2%, respectively). The low prevalence of obesity among these park workers was due to the large number of respondents who partook in regular exercises or were involved in regular physical activity; despite the coronavirus outbreak, this is collaborated by reports that show that physical activity levels are a major influence on obesity (Bülbül 2019). Another similar study carried out by Anto et al. (Anto et al. 2020) reported that the body mass index value of bus drivers was in the normal health category. However, 16% were overweight and 15% were underweight.

From the study carried out, cereal contributes to 50 to 60% of the daily calorie intake of the respondents, and rice is the major food consumed in the cereal/grains group on a higher percentage. According to a study carried out, cereals continue to remain by far the most important food source in the world, contributing 50 percent of calories and as much as 54 percent in developing countries. Their contribution to energy intake varies markedly between developing and industrial countries, and cereals can also contribute as much as 70 percent of energy intake (Sharma et al. 2020).

There was high consumption of tubers among the respondents, but majorly cassava products were the highly consumed root and tubers, while a lower percentage of the respondents consume potatoes compared to a national study on food consumption which reported that the consumption of potatoes rose from 25 to 96 g per capita per day over the same time. Contrasting patterns in the consumption of potatoes may be seen between industrial (falling levels) and developing countries (rising levels). Beef is the one meat category that on a worldwide level showed no growth in consumption levels during this time. This trend shows the fact that while beef consumption grew modestly in certain regions (in developing nations such as China and Brazil), it fell very modestly in most other regions (North America, Oceania, and Europe). A 2050 forecast suggests that the consumption of meat will increase moderately, and this will largely reflect an increase in pork and particularly poultry (OECD and FAO 2018). In this study, there was high consumption of beef meat among the respondents and low consumption of pork meat.

The majority of the respondents from the study do consume fish, and fish is consumed more than once a day by the majority (80%) of the respondents. While fish catchers worldwide are on the increase, the total food fish supply and hence consumption have been growing at a rate of 3.6% per year since 1961, and the world’s population has been expanding at 1.8% per year (Rahman and Islam 2020). The proteins derived from fish, crustaceans, and mollusks account for between 13.8 and 16.5% of the animal protein intake of the human population.

A decreasing trend in milk consumption has been observed in Spain in recent years. A significant decrease in the number of purchased dairy products has taken place from the years 2000 (416 g/day) to 2012 (359 g/day), and one of the main concerns is the possibility that milk is being replaced by other less nutrient-dense foods and beverages, mainly in younger age groups (Maillot et al. 2019). In this study, there was a decrease in the trend of consumption of dairy products. Milk is slightly consumed more than other dairy products like cheese, yogurt, etc.

This study shows a high prevalence of alcohol consumption, 69% of the respondents do consume alcohol, and 63.5% do consume it on daily bases, while 8.0% of the respondents consume alcohol just on a feast day. The clinical interview carried out on AUD revealed that most of the respondents had intermediate AUD (44%). The majority (95%) of the drivers that consume alcohol do consume beer. A similar study recorded a high prevalence of consumption of alcohol (57.9%) and current cigarette smoking (20.6%) among participants (Rautela et al. 2019). Another similar study carried out in Ile-Ife shows the prevalence of alcohol consumption; in the study population, 67.2% were reported to consume alcohol, although the majority of the users reported drinking after the days’ work (Skewes and Lewis 2016). Several other similarities were found between this study and others on the pattern of alcohol use.

The mean energy intake of the respondents was 2788.70 kcal with a recommended daily allowance percentage of 111.54% exceeding the daily requirement of 2500 kcal for an adult male. The Food Consumption Survey (FCS) conducted in Spain in 1987 revealed that the mean energy consumption for the Spanish adult population in 2010 was 2609 kcal/person/day, which was lower than in 1964 (3008 kcal/person/day) (Ruiz et al. 2015) (Varela-Moreiras et al. 2013).

From the study carried out, there was a high intake of dietary fiber among the respondents compared to a study that reported low fiber intake among middle-aged people in several states in the USA (Park et al. 2011). The mean iron intake of the respondents was higher than the RDA accounting for 201% of the RDA percentage. An earlier study by Harrison-Findik (Harrison-Findik 2007) found that iron stores increased progressively across classes of alcohol intake in heavy drinkers and people with AUD. Even in volunteers, drinking small amounts of alcohol compared with teetotalers, there was a significant increase in indices of iron stores, such as ferritin (Raka et al. 2020).

A previous study has found that the consumption of ethanol contributes to daily energy intake significantly (Fawehinmi et al. 2012). From the study carried out, 22.07% of alcohol content in alcoholic beer contributes to a mean intake of 407.63 g of carbohydrates. Men are also more likely to drink beer, which is rich in carbohydrates and provides more energy than wine per standard drink. The nutritional state of an individual may influence the utilization of the energy derived from alcoholic beverages. It is possible that, in humans, lean individuals have a more inefficient utilization of ethanol calories, and that in obese individuals the calories contribute to an increase in body mass (Camacho and Ruppel 2017). Previous studies have found that increased consumption of ethanol increases total daily energy intake significantly (Kwok et al. 2019). Contrary to this study, the focus of a study was to explore the dietary intake of respondents, where it was observed that total energy intake across the four levels of total ethanol intake for individuals with a form of AUD was below the minimum recommended energy intake for both males and females (Fawehinmi et al., 2012).

Alcoholic beer consumed by the respondents contributes to some nutrient intake by the respondents. Fiber from alcohol contributes 9.3% of 31.93 g, and vitamin B1 from alcoholic beer contributes 8.42% of 3.65 mg of vitamin B1 intake. Potassium content contributes to 24.5% of the mean potassium intake of 1805.77 mg. From the study carried out, it was observed that 33–60 cl of beer consumed by the respondents do not show a significance on the respondents’ nutritional status, and also, most of the respondents consume between 30 and 40 ml of ethanol which is slightly significant on the respondents’ body mass index. Studies relating to nutritional status and alcohol intake observed that despite their higher alcohol intake, drinkers were no more obese than nondrinkers and concluded that alcohol calories may be less efficiently utilized (Traversy and Chaput 2015).

## Conclusion

The COVID‑19 pandemic and its related government attempt to reduce movement influenced patterns of alcohol consumption. While the path to recovery remains long and difficult, this crisis also increases the risk that individuals engage in harmful drinking to cope with stress. Commercial drivers operating in Ogun state's motor parks have a high prevalence of alcohol consumption because it is typically sold within their motor parks. There was no significant relationship established between respondents' alcohol consumption and nutritional status. Nonetheless, alcohol and alcoholic beverage consumption should be a public health issue because it may potentially cause consumers to develop chronic diseases, as well as being one of the leading causes of road accidents in the country.

## Data Availability

All data will be made available upon reasonable request.

## Abbreviations

AUD: Alcohol use disorder
CMV: Commercial motor vehicles
RDA: Recommended Dietary Allowance
TDA: Total Dietary Assessment
WHO: World Health Organization
FCS: The Food Consumption Survey
FFQ: Food Frequency Questionnaire
DSM-5: The Fifth Edition of the Diagnostic and Statistical Manual of Mental Disorders
